# Use of proton pump inhibitors is associated with increased risk of out-of-hospital cardiac arrest in the general population: a nested case-control study

**DOI:** 10.1093/ehjcvp/pvae020

**Published:** 2024-03-14

**Authors:** Talip E Eroglu, Ruben Coronel, Gunnar H Gislason

**Affiliations:** Department of Cardiology, Copenhagen University Hospital—Herlev and Gentofte, Gentofte Hospitalsvej 6, PO Box 635, DK-2900 Hellerup, Denmark; Division of Pharmacoepidemiology and Clinical Pharmacology, Utrecht Institute for Pharmaceutical Sciences, Utrecht University, 3584 CS Utrecht, The Netherlands; Amsterdam UMC, Academic Medical Center, University of Amsterdam, Department of Experimental and Clinical Cardiology, Heart Centre, Amsterdam Cardiovascular Sciences, Meibergdreef 9, 1105 AZ Amsterdam, The Netherlands; Department of Cardiology, Copenhagen University Hospital—Herlev and Gentofte, Gentofte Hospitalsvej 6, PO Box 635, DK-2900 Hellerup, Denmark; The Danish Heart Foundation, Department of Research, Vognmagergade 7, DK-1120 Copenhagen, Denmark

**Keywords:** Proton pump inhibitors, Pharmaco-epidemiology, Sudden cardiac arrest

## Abstract

**Aims:**

Proton pump inhibitors (PPIs) impair cardiac repolarization, prolong the QT interval, and may potentially be pro-arrhythmic. However, the risk of out-of-hospital cardiac arrest (OHCA) is scarcely investigated. We studied whether past or current PPI use is associated with OHCA in the general population.

**Methods and results:**

We conducted a nationwide nested case-control study with OHCA-cases of presumed cardiac causes and age/sex/OHCA-date-matched non-OHCA-controls from the general population. Exposure to PPI was categorized into three mutually exclusive groups of current-, past-, and non-use. Conditional logistic regression analyses with adjustments for risk factors of OHCA were used to calculate the odds ratio (OR) of OHCA comparing PPI use with non-users. We identified 46 578 OHCA cases and 232 890 matched non-OHCA controls (mean: 71 years, 68.8% men). PPI was used by 8769 OHCA-cases and 21 898 non-OHCA controls, and current use of PPI was associated with increased odds of OHCA compared with non-users [OR: 1.32 (95% CI: 1.28–1.37)], while past use conferred no increase in the odds of OHCA [OR: 1.01 (95% CI: 0.98–1.04)]. This increased odds of OHCA occurred in both sexes. Finally, the ORs remained elevated when we repeated the analyses in individuals without registered ischaemic heart disease [OR: 1.36 (95% CI: 1.31–1.41)], without heart failure [OR: 1.33 (95% CI: 1.29–1.38)], or without any cardiovascular comorbidities [OR: 1.84 (95% CI: 1.70–2.00)]. Also, the OR remained elevated when H_2_-antagonists served as the reference group [OR: 1.28 (95% CI: 1.11–1.47)].

**Conclusion:**

PPI use is associated with an increased risk of OHCA in the general population. Considering the widespread use of PPIs, this study raises concerns and the need for awareness to balance the benefit and risk of treatment.

## Introduction

Out-of-hospital cardiac arrest (OHCA) accounts for 50% of all cardiovascular deaths in industrialized countries,^1^ and is usually caused by cardiac arrhythmias (ventricular tachycardia/ventricular fibrillation) that occur predominantly in the setting of structural heart disease.^2^ Many commonly prescribed drugs may impact cardiac electrophysiology and increase the risk of OHCA; this applies both to drugs prescribed for cardiac disease and drugs for non-cardiac disease (e.g. antipsychotics, antidepressants, and antibiotics).^3,4^ The best-known mechanism by which drugs increase the risk of OHCA is by blocking cardiac potassium channels, thereby impairing cardiac repolarization and prolonging the QT interval on the electrocardiogram, potentially leading to Torsade de Pointes (TdP) arrhythmia.^5^

Proton pump inhibitors (PPIs) are widely used to treat gastroesophageal reflux disease and other related disorders. Users of PPIs are at increased risk of cardiovascular mortality and comorbidity^6–9^ and previous studies suggested that higher risk of cardiovascular death associated with PPI use might be, at least in part, due to an increased risk of life-threatening ventricular arrhythmias based on TdP arrhythmias.^10,11^ Indeed, it has been reported that PPIs have a direct impact on cardiac electrophysiology by blocking cardiac potassium channels, leading to impaired cardiac repolarization and QT prolongation.^12–14^ Accordingly, this may increase the susceptibility to triggered cardiac arrhythmias by early afterdepolarizations in users of PPIs. We hypothesize that PPIs increase the risk of OHCA. To our knowledge, no data are available on the risk of OHCA upon use of PPIs. Filling this knowledge gap is needed given that the prescription of PPIs has increased rapidly during the past decades, and PPIs are among the most frequently prescribed drugs worldwide.^15,16^ Our aim of the current study was therefore to establish whether PPI use is associated with an increased risk of OHCA using nationwide data from a large cohort of OHCA victims that was specifically designed to study OHCA in the general population.

## Methods

### Ethics

The study has been approved by the Danish Data Protection Agency (Ref. no. 2007-58-0015, local ref. no. GEH-2014-017, I-Suite 0.2735). In Denmark, no further ethical approval or informed consent is required for register-based studies where patients remain anonymous.

### Study design and population

This nested case-control study was conducted using a nationwide cohort of individuals in the period between 1 June 2001 and 31 December 2019 from the Danish health registries. The cases were OHCA victims of a presumed cardiac cause from The Danish Cardiac Arrest Registry (DANCAR). Patients who suffered OHCA from non-cardiac causes (e.g. drug intoxication/overdose, drowning, trauma, and other non-cardiac diseases) were excluded. At the index date (date of OHCA or control date), each OHCA case was randomly matched by sex and birth year in a 1:5 ratio to controls without OHCA that were drawn from the general population. This study approach and study population has been previously used by this research group.^17^

### Data sources

Residents in Denmark have a unique and permanent identification number that enables individual-level linkage between nationwide registries on the national healthcare system, which allows us to conduct large-scale research with nationwide coverage. The five nationwide registers that were linked and used for this study are described below:

1. The Danish National Population Register, which contains information concerning date of birth, gender, and immigration and emigration status.
2. The Danish National Patient Register, which holds information on all admissions to Danish hospitals since 1978 with diagnoses and procedures coded according to the International Classification of Diseases 10th revision (ICD-10).
3. The National Prescription Registry, which contains complete drug-dispensing records dispensed in pharmacies since 1995 that is classified according to the Anatomical Therapeutic Chemical (ATC) system.
4. The National Causes of Death Registry, where the causes of death in Denmark are registered.
5. The DANCAR, which is a nationwide registry containing information on all OHCAs with a resuscitation attempt occurring in Denmark since June 2001. OHCA has been defined as a clinical condition of unresponsiveness where an ambulance has been summoned and where cardiopulmonary resuscitation has been attempted, either by a bystander or emergency medical service (EMS) personnel. The capture of OHCA cases is nearly complete because the EMS is activated for all clinical emergencies in Denmark, and EMS personnel must complete a case report for every attended OHCA. The presumed cause of OHCA was obtained from the death certificates and discharge diagnosis codes. OHCAs of presumed cardiac cause were events with diagnosis codes for cardiac disease, unknown disease, or unexpected collapse (and were included in the present study).

### Exposure of interest and covariates

The exposure of interest was the use of PPIs, which were categorized into three mutually exclusive groups of current-, past-, and non-use. Current use of PPIs was defined as any dispensed prescription within 90 days prior to the index date using the ATC code A02BC. Past use of PPIs was defined as any redeemed prescription before the 90 days prior to index date. If patients had no prescription for any type of PPI prior to the index date they were considered as non-exposed.

Next, the following comorbidities were identified in a 10-year lookback period from the index date: ischaemic heart disease, heart failure, atrial fibrillation, cerebrovascular disease, peripheral artery disease, severe psychiatric disorders (schizophrenia, schizotypal, delusional, and non-mood psychotic disorders), depression, chronic obstructive pulmonary disease, and chronic kidney disease. Since the diagnosis code of diabetes mellitus has a low sensitivity in the Danish registries, diabetes mellitus was identified using drug-dispensing records, and its presence was defined as a claimed prescription for an oral glucose-lowering drug within 180 days prior to index date. Also, hypertension was identified using drug-dispensing records in which a redeemed prescription of two or more blood pressure-lowering drugs prior to the index date was used as a proxy for hypertension, as done previously.^18^ By doing this, patients being treated for hypertension and diabetes mellitus outside of hospitals (e.g. general practice) could be included. Finally, concomitant pharmacotherapy was defined as having a drug-dispensing record for any of the following medications within 180 days before the index date: beta-blockers, calcium-channel blockers, antithrombotics, diuretics, renin-angiotensin system inhibitors, nitrates, Vaughan-Williams class I or III antiarrhythmic drugs, non-steroidal anti-inflammatory drugs (NSAIDs), inhalation medication used in the treatment of chronic obstructive pulmonary disease (inhaled corticosteroids, long-acting beta-2-agonists, long-acting muscarinic antagonists), and QT-interval prolonging drugs. Drugs with QT-interval-prolonging properties (see Supplementary material online, *Table S1* for an overview) were obtained from the CredibleMeds list,^19^ as done previously.^17^ For QT-interval prolonging drugs that are being prescribed for shorter periods, such as antibiotics, the exposure time was shortened to 14 days before the index date. An overview of all the used ICD-10 and ATC codes is provided in Supplementary material online, *Table S1*.

### Statistical analyses

Conditional logistic regression models were used to estimate odds ratios (ORs) and corresponding 95% confidence intervals (95% CIs) of OHCA comparing PPI use with non-users. The models were adjusted for the following pre-specified well-known risk factors of OHCA: ischaemic heart disease, heart failure, atrial fibrillation, cerebrovascular disease, peripheral artery disease, chronic obstructive pulmonary disease including inhalation medication used in the treatment of chronic obstructive pulmonary disease, diabetes mellitus, hypertension, chronic kidney disease, number of cardiovascular drugs, use of NSAIDs, and the use of QT-prolonging drugs. We further studied the relation between PPIs and OHCA by stratifying according to sex to investigate a potential effect modification. The presence of interaction on a multiplicative scale between PPIs and sex was estimated by consecutively including the cross-product of the two factors as a variable in the model. A two-tailed *P*-value of <0.05 was considered indicative of a significant difference among groups. Finally, three pre-specified sensitivity analyses were conducted to assess the robustness of the results. First, the analyses were repeated using an alternative comparator in which the non-exposed category consisted of current users of H_2_-receptor antagonists, which were defined similar as PPIs. Second, we repeated our analyses in subjects without ischaemic heart disease, without heart failure, without any of the cardiovascular comorbidities, or in patients with the presence of cardiovascular comorbidities. Third, the primary analyses were repeated by taking different exposure windows to define the current use of PPIs (i.e. 30 days, 14 days instead of 90 days).

## Results

### Population characteristics

We identified 46 578 cases with OHCA and matched them with 232 890 controls without OHCA from the general population. Characteristics of the included cases and controls are shown in Supplementary material online, *Table S1*. Due to matching, sex and age composition were identical for OHCA cases and non-OHCA controls, with 66.8% of patients being men at a mean age of 71 years (SD 14.40). As expected, the overall comorbidity burden was higher for OHCA cases than for non-OHCA controls. Also, the use of cardiovascular drugs and QT-prolonging drugs was more prevalent among cases than their matched controls. *Table 1* presents the characteristics of OHCA cases stratified according to PPI use. Current users of PPIs were older, with a higher proportion of women compared to non-users. Furthermore, the overall comorbidity burden and concomitant drug use were higher for current users of PPIs compared to non-users.

**Table 1:** Characteristics of out-of-hospital cardiac arrest cases stratified according to current-, past-, and non-use of proton pump inhibitors

	Cases with current PPI use (*n* = 8769)	Cases with past PPI use (*n* = 10 862)	Cases without PPI use (*n* = 26 947)
Age (years), median [IQR]	75 [66,83]	74 [63,82]	71 [61,80]
Male sex, *n* (%)	5285 (60.27)	7062 (65.02)	18 784 (69.71)
Comorbidity, *n* (%)			
Ischaemic heart disease^a^	3467 (39.54)	3372 (31.04)	5228 (19.40)
Heart failure	2651 (30.23)	2578 (23.73)	4230 (15.70)
Atrial fibrillation	2270 (25.89)	2350 (21.64)	3944 (14.64)
Diabetes mellitus	1978 (22.56)	1867 (17.19)	3566 (13.23)
Cerebrovascular disease	1779 (20.29)	1807 (16.64)	2956 (10.97)
Peripheral artery disease	1574 (17.95)	1554 (14.31)	2277 (8.45)
Severe psychiatric disorders	355 (4.05)	347 (3.19)	628 (2.33)
Depression	779 (8.88)	781 (7.19)	882 (3.27)
Chronic obstructive pulmonary disease	2248 (25.64)	2060 (18.97)	2781 (10.32)
Chronic kidney disease	1208 (13.78)	1020 (9.39)	1051 (3.90)
Hypertension	1571 (17.92)	1736 (15.98)	4454 (16.53)
Concomitant pharmacotherapy, *n* (%)			
Beta blockers	3013 (34.36)	3016 (27.77)	5691 (21.12)
Calcium channel blockers	2141 (24.42)	2358 (21.71)	4941 (18.34)
Antithrombotics	5359 (61.11)	5577 (51.34)	10 794 (40.04)
Diuretics	5600 (63.86)	5621 (51.75)	11 599 (43.04)
Renin-angiotensin system inhibitors	3912 (44.61)	4464 (41.10)	9541 (35.41)
Nitrates	1522 (17.36)	1271 (11.70)	2080 (7.72)
Antiarrhythmic drugs class 1 or 3	277 (3.16)	248 (2.28)	393 (1.46)
Non-steroidal anti-inflammatory drugs	1681 (19.17)	1702 (15.67)	3751 (13.92)
Chronic obstructive pulmonary disease treatment			
Inhaled corticosteroids	1626 (18.54)	1517 (13.97)	2418 (8.97)
Long-acting beta-2 agonists	2404 (27.41)	2298 (21.16)	3642 (13.52)
Long-acting muscarinic antagonists	1654 (18.86)	1497 (13.78)	2182 (8.10)
QT-interval prolonging drugs	2269 (25.88)	2007 (18.48)	3147 (11.68)
Number of cardiovascular drug use			
0	1147 (13.08)	2592 (23.86)	9505 (35.27)
1	1343 (15.32)	1669 (15.37)	4089 (15.17)
2	1820 (20.75)	2107 (19.40)	4653 (17.27)
>2	4459 (50.85)	4494 (41.37)	8700 (32.29)

Numbers are number (%) unless indicated otherwise.

PPI, proton pump inhibitor.

a Including acute myocardial infarction.

### Association between PPIs and OHCA

*Table 2* presents the results related to OHCA. Within 90 days prior to the index date, PPI was currently used by 8769 (18.83%) OHCA cases and 21 898 (9.40%) non-OHCA controls, and current use of PPI was associated with increased odds of OHCA compared with no use of PPI after adjusting for all the relevant confounders in our multivariate analyses [adjusted OR 1.32 (95% CI: 1.28–1.37), *Table 2*], with pantoprazole having the highest odds of OHCA [adjusted OR 1.52 (95% CI: 1.45–1.60)], while past use of PPI conferred no increase in the odds of OHCA [adjusted OR 1.01 (95% CI: 0.98–1.04), *Table 2*]. The odds of OHCA were highest in recent starters (<90 days) of PPIs [adjusted OR 2.85 (95% CI: 2.60–3.11)]. Stratification according to sex showed that increased odds of OHCA were present in both men and women [adjusted OR_Male_ 1.32 (95% CI: 1.26–1.37), adjusted OR_Female_ 1.32 (95% CI: 1.25–1.39), *P*-value interaction: 0.991, *Table 2*). Further, our sensitivity analyses yielded consistent findings (*Table 3*), where the ORs remained elevated when we repeated the analyses in individuals without registered ischaemic heart disease [adjusted OR 1.36 (95% CI:1.31–1.41)], without heart failure [adjusted OR 1.33 (95% CI: 1.29–1.38)], without any cardiovascular comorbidities [adjusted OR 1.84 (95% CI: 1.70–2.00)], or in patients with the presence of cardiovascular comorbidities [adjusted OR 1.39 (95% CI:1.34–1.43)]. Also, the OR remained elevated when H_2_-antagonists served as the reference group [adjusted OR 1.28 (95% CI: 1.11–1.47)]. Finally, the ORs did not vary when analyses were conducted by using different exposure windows to define current users of PPIs [adjusted OR_30 days_ 1.43 (95% CI: 1.37–1.49), adjusted OR_14 days_ 1.51 (95% CI: 1.43–1.59)].

**Table 2:** Association between the use of proton pump inhibitors and the odds ratio of out-of-hospital cardiac arrest

	Cases (*n* = 46 578)	Controls (*n* = 232 890)	Adjusted OR (95% CI)^a^
No use of PPI	26 947 (57.85)	164 006 (70.42)	1.0 (reference)
Past use of PPI	10 862 (23.32)	46 986 (20.18)	1.01 (0.98–1.04)
Current use of PPI	8769 (18.83)	21 898 (9.40)	1.32 (1.28–1.37)
Omeprazole	1541 (3.31)	5038 (2.16)	1.15 (1.08–1.23)
Pantoprazole	3978 (8.54)	7969 (3.42)	1.52 (1.45–1.60)
Lansoprazole	1885 (4.05)	5828 (2.50)	1.14 (1.07–1.21)
Esomeprazole	1071 (2.30)	2519 (1.08)	1.34 (1.23–1.45)
Rabeprazole	31 (0.07)	116 (0.05)	1.12 (0.73–1.71)
≥2 PPI	263 (0.56)	428 (0.18)	1.84 (1.54–2.19)
Versus H_2_-receptor antagonist			
Current use of H_2_-receptor antagonist	307 (0.66)	1243 (0.53)	1.0 (reference)
Current use of PPI	8769 (18.83)	21 898 (9.40)	1.28 (1.11–1.47)
Duration of use in current users			
No use of PPI	26 947 (57.85)	164 006 (70.42)	1.0 (reference)
≤90 days	1045 (2.24)	1469 (0.63)	2.85 (2.60–3.11)
>90 days	7724 (16.58)	20 429 (8.77)	1.22 (1.18–1.26)
Sex stratified (*P*-value interaction: 0.991)			
Men	31 131	155 655	
No use of PPI	18 784 (60.34)	113 290 (72.78)	1.0 (reference)
Past use of PPI	7062 (22.68)	29 369 (18.87)	1.03 (0.99–1.07)
Current use of PPI	5285 (16.98)	12 996 (8.35)	1.32 (1.26–1.37)
Women	15 447	77 235	
No use of PPI	8163 (52.85)	50 716 (65.66)	1.0 (reference)
Past use of PPI	3800 (24.60)	17 617 (22.81)	0.98 (0.93–1.02)
Current use of PPI	3484 (22.55)	8902 (11.53)	1.32 (1.25–1.39)

Numbers in table are number (%) unless indicated otherwise.

CI, confidence interval; OR, odds ratio; PPI, proton pump inhibitor.

a Adjusted for ischaemic heart disease, heart failure, atrial fibrillation, diabetes mellitus, cerebrovascular disease, periphery artery disease, severe psychiatric disorders, depression, chronic obstructive pulmonary disease including chronic obstructive pulmonary disease treatment, chronic kidney disease, number of cardiovascular drugs, use of non-steroidal anti-inflammatory drugs and use of QT-interval prolonging drugs.

**Table 3:** Association between the use of proton pump inhibitor and the odds ratio of out-of-hospital cardiac arrest in individuals with the absence of (1) ischaemic heart disease, (2) heart failure, (3) any cardiovascular comorbidities, or (4) in patients with the presence of cardiovascular comorbidities

Absence ischaemic heart disease			
	**Cases (*n* = 34 511)**	**Controls (*n* = 208 788)**	**Adjusted OR (95% CI)**
No use of PPI	21 719 (62.93)	152 949 (73.26)	1.0 (reference)
Current use of PPI	5302 (15.36)	16 728 (9.01)	1.36 (1.31–1.41)
**Absence heart failure**			
	**Cases (*n* = 37 119)**	**Controls (*n* = 222 772)**	**Adjusted OR (95% CI)**
No use of PPI	22 717 (61.20)	159 288 (71.50)	1.0 (reference)
Current use of PPI	6118 (16.48)	19 538 (8.77)	1.33 (1.29–1.38)
**Absence cardiovascular comorbidities**			
	**Cases (*n* = 11 367)**	**Controls (*n* = 117 591)**	**Adjusted OR (95% CI)**
No use of PPI	8390 (73.81)	96 877 (82.38)	1.0 (reference)
Current use of PPI	883 (7.77)	4460 (3.79)	1.84 (1.70–2.00)
**Presence cardiovascular comorbidities**			
	**Cases (*n* = 35 211)**	**Controls (*n* = 115 299)**	**Adjusted OR (95% CI)**
No use of PPI	18 557 (52.70)	67 129 (58.22)	1.0 (reference)
Current use of PPI	7886 (22.40)	17 438 (15.12)	1.39 (1.34–1.43)

Numbers in table are number (%) unless indicated otherwise. Numbers and ORs for past users have not been included in this table.

CI, confidence interval; OR, odds ratio; PPI, proton pump inhibitor.

## Discussion

In this nationwide study, including data from more than 45 000 OHCAs of presumed cardiac cause in Denmark, current PPI use was associated with significantly increased odds of OHCA after adjustment for relevant OHCA risk factors in the general population. This increased odds of OHCA occurred in both sexes. Further, our finding of increased odds of OHCA associated with PPIs remained consistent in subjects without the presence of cardiovascular comorbidities. Finally, our results were consistent across several sensitivity analyses in which H_2_-antagonists served as the reference group or when different exposure windows were used to define current users of PPIs.

Our results indicate that the prevalence of risk factors that increase OHCA risk, in particular, cardiovascular comorbidities, was higher among users of PPIs compared to non-users of PPIs. This is in line with previous studies in which increased risk of cardiovascular comorbidities associated with PPI use have been demonstrated.^8,9^ This might result in bias since cardiovascular comorbidities are closely related to OHCA.^2^ Consequently, it cannot be ruled out that these variables may have contributed to the relationship between PPI and OHCA in our study. Nevertheless, current use of PPIs remained significantly associated with increased odds of OHCA after adjustment for the presence of these variables in our multivariate model. Moreover, our finding of increased odds of OHCA remained consistent in subjects without the presence of cardiovascular comorbidities, suggesting that the link between PPIs and OHCA is not explained, at least not solely, by differences in these traditional risk factors. Our data therefore suggest a direct impact of PPI on OHCA. Indeed, it has been hypothesized that PPIs may impact cardiac electrophysiology and increase the risk of OHCA due to several mechanisms.^10–14^ First, PPIs may have a direct interference on the electrophysiological properties of the cardiomyocyte by causing electrolyte abnormalities through reducing the circulating magnesium levels.^10,13^ In a recent meta-analysis, hypomagnesaemia was found in 19.4% of PPI users.^20^ Although the exact mechanism of PPI-induced hypomagnesaemia is not fully understood, the pathogenesis probably includes both gastrointestinal and renal losses.^21^ Magnesium plays an important role in regulating cardiac potassium and calcium channels that are important in controlling the heart's electrical properties.^22^ Experimental studies have demonstrated that cytosolic magnesium promotes repolarization of myocardial cells via modulating effects on several cardiac potassium currents, including the rapid component of the delayed rectifier potassium current (I_Kr_) and the transient outward current (I_to_).^23,24^ This in turn may lead to action potential prolongation and triggered activity based on early after depolarizations. Further, magnesium may affect intracellular calcium by blocking the L-type calcium channel pore.^25^ Correspondingly, PPI-induced hypomagnesaemia could increase the cytoplasmic Ca^2+^ concentrations in cardiomyocytes, which may increase the risk of cardiac arrhythmias and OHCA by provoking delayed afterdepolarizations as well as by increasing the action potential duration. Also, recent data suggests that PPIs may impact cardiac electrophysiology regardless of hypomagnesaemia by directly blocking the ether-a-go-go-related gene potassium channel (hERG) current.^12–14^ Lorberbaum et al. showed that clinically relevant concentrations of lansoprazole caused a reduction of hERG current and prolonged the QT interval when used in combination with ceftriaxone.^14^ Moreover, cases with prolonged QT intervals and TdP in the absence of low magnesium levels have been reported during PPI treatment.^26,27^ Supporting this, Lazzerini et al. showed that in the majority of the PPI-treated individuals (∼60%), TdP developed in the presence of normal magnesium levels.^11^ In line with this, using a database of patients admitted to an intensive care unit, Fan et al. showed a prolonged QT interval associated with PPI treatment, which was considered to be independent of known QT-prolonging factors.^28^ Finally, in another study, Lazzerini et al. investigated the effect of PPIs on hERG currents *in vitro* and then reviewed the impact of PPIs on the risk of QT prolongation using data from a US Veterans Administration database.^12^ In that study, it was demonstrated that clinically relevant concentrations of different PPIs induced a concentration-dependent inhibition of the hERG current *in vitro*, with pantoprazole being the most potent compound^12^; this is also in line with our results, in which current users of pantoprazole had the highest OHCA risk. Further, in the US veterans cohort, it was reported that PPI treatment was independently associated with about a 20%–40% increased risk of QT prolongation, which was independent of their magnesium-lowering effect.^12^

We also studied the associations of each individual PPI separately. Our findings indicate that it is likely that the OHCA-risk-increasing effects of PPIs are a class effect with the highest risk among users of pantoprazole. Although we did not find a statistically significant association between current use of rabeprazole and OHCA in our study, a trend towards increased risk was observed. It should be noted, however, that the number of rabeprazole users was low in our study, which is also highlighted by the wide confidence interval. It is therefore likely that a lack of statistical power masked an otherwise significant association. Correspondingly, our findings related to rabeprazole need to be considered carefully. Further, patients who redeemed prescriptions for different PPIs within 90 days prior to index date were classified as combined users in our analyses since it was difficult to define exactly which PPI they used. It is, however, possible that these patients switched from one PPI to another PPI rather than a combined use.

We also stratified our analysis according to sex, expecting that the OHCA risk associated with the use of PPI would be larger in women than in men, considering that women have less repolarization reserve than men and are therefore more susceptible to QT prolongation than men.^29^ We found, however, a similarly increased OHCA risk associated with the use of PPIs in men and women. Further, the OHCA risk associated with PPI use remained elevated when we performed our analyses in a subset of patients without pre-existing cardiovascular comorbidities, thereby providing additional support for the notion that the observed association in the present study was due to a drug effect.

Our study adds important data to the discussion concerning the risk of potentially fatal cardiac arrhythmias of PPIs. This is of clinical importance given the widespread use of PPIs. Further studies in other large-scale registries with OHCA victims that are well-phenotyped in a uniform manner are warranted to confirm our findings; our study may provide the basis for future research.

### Strengths and limitations

A major strength of our study is its population-based real-world design, in which a large and unselected number of OHCA victims were included from nationwide registries, thereby minimizing the risk of selection bias by including every OHCA prospectively and increasing the likelihood that our findings are applicable to the community at large. Furthermore, since the DANCAR registry was specifically designed to study OHCA in the general study, it was possible to include both patients who survived hospital admission and those who died prehospital, thereby minimizing the risk of inclusion bias in our study. Nevertheless, as with any observational study, our study has some limitations. First, it was not possible to obtain information on lifestyle factors (e.g. smoking status, alcohol use, body mass index), and therefore these could not be taken directly into account. This could lead to bias because PPI users have been shown to have an unhealthier lifestyle than non-users of PPI.^30^ Nonetheless, we adjusted for important consequences related to an unhealthy lifestyle (e.g. ischaemic artery disease, heart failure, diabetes mellitus, and chronic obstructive pulmonary disease) in the multivariate analyses. Still, residual confounding could not be ruled out, especially if unmeasured risk factors for OHCA (e.g. lifestyle-related) were more prevalent in PPI users compared to non-users. Second, it was not possible to obtain information regarding electrolyte abnormalities (e.g. hypokalaemia) and therefore it was not possible to adjust for electrolyte abnormalities in our multivariate analyses. Third, misclassification of the exposure may have occurred due to Pro re nata use (taking the drug when needed), since data on pharmacotherapy was based on drug-dispensing records without further information on actual drug intake. However, it is likely that a possible misclassification arising from this was probably similarly distributed between cases and controls. Five, data regarding over-the-counter use and in-hospital intravenous use of PPI were not recorded and therefore not available, leading to underestimating PPI use and a possible misclassification of PPI users as non-users. However, in Denmark, only around 2% of the total PPI use is bought over-the-counter, which allowed us to estimate PPI exposure based on redeemed prescriptions accurately.^31^ It is therefore unlikely that this limitation impacted the interpretation or generalizability of our findings.

## Conclusion

Use of PPI is associated with an increased risk of OHCA in the general population and appears to be independent of sex. Furthermore, this increased OHCA risk associated with PPIs remained consistent in subjects without the presence of cardiovascular comorbidities. Considering the widespread use of PPIs, this study raises concerns and the need for awareness to balance the benefit and risk of treatment and to mitigate the risk of OHCA.

## Supplementary Material

pvae020_Supplemental_File

## Data availability

The data underlying this article cannot be shared publicly due to ethical/privacy reasons.
