# Supplementary material for: Use of proton pump inhibitors is associated with increased risk of out-of-hospital cardiac arrest in the general population: a nested case-control study
Source: Eur Heart J Cardiovasc Pharmacother. 2024 Mar 14;10(5):413–9. doi: 10.1093/ehjcvp/pvae020 (PMC11323370; doi:10.1093/ehjcvp/pvae020)
Supplement: pvae020_Supplemental_File [file pvae020_supplemental_file.docx]

**Supplementary material**

**Supplementary Table 1. ICD-10 codes, procedure and ATC codes used throughout the study**

| Disease | ICD-10 codes | Procedure codes | ATC codes |
| --- | --- | --- | --- |
| Ischemic heart disease† | I20-I25 | KFNA, KFNB, KFNC, KFND, KFNE, KFNG, KFNF, KFNH20 |  |
| Heart failure | I50, I110, I130, I132, I420, I426-I429, I421, I422, I425 |  |  |
| Atrial fibrillation | I48 |  |  |
| Diabetes mellitus |  |  | A10 |
| Cerebrovascular disease | I60-I69, G45-G46 |  |  |
| Peripheral artery disease | I70-I74, I77 |  |  |
| Severe psychiatric disorders (schizophrenia, schizotypal, delusional, and non-mood psychotic disorders) | F20-F29 |  |  |
| Depression | F32, F33 |  |  |
| COPD | J42-J44 |  |  |
| Chronic kidney disease | N02-N08, E102, E112, E132, E142,  I120, Q612, Q613, Q615, Q619,  N158, N159, N160, N162, N163,  N164, N168, M300, M313, M319,  N11, N12, N14, N18, N19, N26,  M321B | BJFD2 |  |
| Hypertension |  |  | α-adrenergic blockers (C02A, C02B, C02C), non-loop diuretics (C02DA, C02L, C03A, C03B, C03D, C03E, C03X, C07C, C07D, C08G, C09BA, C09DA, C09XA52), vasodilators (C02DB, C02DD, C02DG, C04, C05), β-blockers (C07), calcium channel blockers (C07F, C08, C09BB, C09DB), renin-angiotensin system inhibitors (C09) |
|  |  |  |  |
| Concomitant pharmacotherapy |  |  |  |
| Beta blockers |  |  | C07 |
| Calcium channel blockers |  |  | C08 |
| Antithrombotics |  |  | B01AA, B01AC, B01AE07, B01AF01, B01AF02, B01AF03 |
| Diuretics |  |  | C03AA, C03AB, C03B, C03C, C03D, C03E, C07B, C07CA, C07CB, C07D, C08G, C09BA, C09DA |
| Renin-angiotensin system inhibitors |  |  | C09A, C09B, C09C, C09D |
| Nitrates |  |  | C01DA |
| Antiarrhythmic drugs class 1 or 3 |  |  | C01B |
| Non-steroidal anti-inflammatory drugs |  |  | M01A |
| COPD treatment |  |  |  |
| Inhaled corticosteroids |  |  | R03BA, R03AK06-R03AK12, R03AL08- R03AL09 |
| Long-acting [beta-2 agonists](https://www.sciencedirect.com/topics/medicine-and-dentistry/beta2-agonist) |  |  | R03AC, R03AK06-R03AK12, R03AL01-R03AL09 |
| Long-acting [muscarinic antagonists](https://www.sciencedirect.com/topics/medicine-and-dentistry/muscarinic-antagonist) |  |  | R03BB, R03AL01-R03AL09 |
| QT-prolonging drugs†† |  |  | Roxithromycin (J01FA06), Azithromycin (J01FA10), Ciprofloxacin (J01MA02), Clarithromycin (J01FA09), Erythromycin (J01FA01), Moxifloxacin (J01MA14), Fluconazole (J02AC01), Levomepromazine (N05AA02), Sulpiride (N05AL01), Sotalol (C07AA07), Quinidine (C01BA01), Pimozide (N05AG02), A04AA01, Ondansetron (N05AD01), Flecainide (C01BC04), Escitalopram (N06AB10), Dronedarone (C01BD07), Donepezil (N06DA02), Domperidone (A03FA03), Citalopram (N06AB04), Droperidol (N05AD08), Chlorpromazine (N05AA01), Chlorprothixene (N05AF03), Amiodarone (C01BD01). |
| † Including acute myocardial infarction  †† QT-prolonging drugs with known risk of Torsade de Pointes according to CredibleMeds website (www.CredibleMeds.org). | | | |

| **Table 1. Study population characteristics** | | |
| --- | --- | --- |
|  | Cases (n=46,578) | Controls  (n=232,890) |
| Age (years), mean [SD] | 71 [14.40] | 71 [14.40] |
| Male sex, n (%) | 31131 (66.84) | 155655 (66.84) |
| **Comorbidity, n (%)** |  |  |
| Ischemic heart disease^§^ | 12067 (25.91) | 24102 (10.35) |
| Heart failure | 9459 (20.31) | 10118 (4.34) |
| Atrial fibrillation | 8564 (18.39) | 16672 (7.16) |
| Diabetes mellitus | 7411 (15.91) | 16975 (7.29) |
| Cerebrovascular disease | 6542 (14.05) | 17717 (7.61) |
| Peripheral artery disease | 5405 (11.60) | 9243 (3.97) |
| Severe psychiatric disorders | 1330 (2.86) | 1897 (0.81) |
| Depression | 2442 (5.24) | 5458 (2.34) |
| COPD | 7089 (15.22) | 10617 (4.56) |
| Chronic kidney disease | 3279 (7.04) | 4867 (2.09) |
| Hypertension | 7761 (16.66) | 22594 (9.70) |
| Obesity | 2395 (5.14) | 4557 (1.96) |
|  |  |  |
| **Concomitant pharmacotherapy, n (%)** |  |  |
| Beta blockers | 11720 (25.16) | 31672 (13.60) |
| Calcium channel blockers | 9440 (20.27) | 33915 (14.56) |
| Antithrombotics | 21730 (46.65) | 60108 (25.81) |
| Diuretics | 22820 (48.99) | 61531 (26.42) |
| Renin-angiotensin system inhibitors | 17917 (38.47) | 55970 (24.03) |
| Nitrates | 4873 (10.46) | 7590 (3.26) |
| Antiarrhythmic drugs class 1 or 3 | 918 (1.97) | 1173 (0.50) |
| Non-steroidal anti-inflammatory drugs | 7134 (15.32) | 28550 (12.26) |
| COPD treatment |  |  |
| Inhaled corticosteroids | 5561 (11.94) | 12707 (5.46) |
| Long-acting [beta-2 agonists](https://www.sciencedirect.com/topics/medicine-and-dentistry/beta2-agonist) | 8344 (17.91) | 16353 (7.02) |
| Long-acting [muscarinic antagonists](https://www.sciencedirect.com/topics/medicine-and-dentistry/muscarinic-antagonist) | 5333 (11.45) | 7733 (3.32) |
| QT-interval prolonging drugs | 7423 (15.94) | 15924 (6.84) |
| Number of cardiovascular drug use |  |  |
| 0 | 13244 (28.43) | 124357 (53.40) |
| 1 | 7101 (15.25) | 32189 (13.82) |
| 2 | 8580 (18.42) | 32997 (14.17) |
| >2 | 17653 (37.90) | 43347 (18.61) |
| Abbreviations: PPI, proton pump inhibitor; COPD, chronic obstructive pulmonary disease | | |
| Numbers are number (%) unless indicated otherwise.  ^§^including acute myocardial infarction | | |
